# Smart Biomimetic 3D Scaffolds Based on Shape Memory Polyurethane for Soft Tissue Repair

**DOI:** 10.3390/polym17070872

**Published:** 2025-03-25

**Authors:** Xiaoling Zuo, Weijing Sun, Yutong Wu, Hanliu Gu, Tao Chen, Ting Zhang, Xiaoying Liu, Jianwei Zhang, Li Wang

**Affiliations:** Department of Biomedical Engineering, School of Big Health and Intelligent Engineering, Chengdu Medical College, Chengdu 610500, China; 13551199054@163.com (X.Z.);

**Keywords:** 3D scaffold, polyurethane, shape memory polymer, microstructure, soft tissue engineering

## Abstract

Tissue-engineered biocompatible scaffolds could mimic the extracellular matrix structure for cell adhesion and proliferation; however, patients suffer from large volume implantation. In this study, a thermal sensitive shape memory polyurethane porous 3D scaffold based on poly(ε-caprolactone) and poly(ethylene glycol adipate) was developed, utilizing the water-splitting property of aliphatic hexamethylene diisocyanate (HDI) to crosslink rigid segments during the polymerization process. The chemical structure, microstructure, and morphology, as well as mechanical strength, of the scaffolds were characterized by Fourier transform infrared spectroscopy (FTIR), X-ray diffraction (XRD), a scanning electron microscope (SEM), and tensile tests. The results show that gas foaming action caused by the release of CO_2_ occurred simultaneously in the reactive process, resulting in the interconnective porous structure of the PU scaffolds with a porosity of over 70% and pore sizes from 100 μm to 800 μm. Additionally, after programming to a temporary shape, the scaffolds could recover to their initial shapes and could be programmed into various shapes according to different defects. These smart shape-changeable scaffolds with high porosity and good physio-chemical properties are a promising material for minimally invasive tissue engineering.

## 1. Introduction

Soft tissues such as cartilage are often difficult to repair once damaged, which can subsequently lead to a series of serious health issues, including pain, swelling, and joint dysfunction, significantly impacting the quality of life for patients [1]. Traditional treatment methods for soft tissue injuries, including microfracture surgery and autologous or allogeneic cartilage transplantation, etc., although capable of alleviating symptoms to some extent, still present numerous limitations and challenges. For example, autologous bone has excellent bone induction properties, bone transmission properties, and osteogenic properties, and is theoretically the most ideal bone graft material, but its source is limited, and it has the disadvantages of damaging other tissues, injuring human nerves, increasing the difficulty of operation, and causing the secondary loss of bone [2]. Allogeneic cartilage transplantation is prone to rejection and potential infectious diseases, and in order to induce osteogenesis between allogeneic bone and self-bone tissue, it is necessary to reduce autoimmunogenicity, leading to low autoimmunity and resulting in poor induction [3]. Artificially synthesized metal materials have excellent fatigue resistance and mechanical properties, and are widely available. However, their compatibility with existing bones in the human body is not ideal, and some metal ions are easily corroded in the human body over time, while others have slight toxicity. In addition, inorganic bioceramics and inorganic bioglass materials with good biological activity and compatibility have been selected as new bone-repair materials. These materials form chemical bonds with the surface of human bones, inducing bone repair and regeneration. However, this type of inorganic material is prone to brittle fracture and poor elasticity. Compared to other materials, the various mechanical properties of polymers are relatively good. Both artificially synthesized materials and natural polymer materials have excellent biodegradability and biocompatibility, so this material has great potential for growth in the field of biomedicine [4]. Shape memory polymers, as a new type of intelligent material, not only have good biocompatibility and degradability, but can also restore a preset shape under specific conditions, providing more stable support for defect repair [5]. Polymer materials with shape memory effects such as polycaprolactone (PCL) and polyurethane (PU) can be selected as the matrix. Polycaprolactone, as an artificially synthesized polymer material, has excellent toughness and certain mechanical properties [6]. In the field of medicine, it has good compatibility, polymerization, and nontoxicity. Rosales Ibáñez et al. used electrospinning technology to prepare polycaprolactone bioactive scaffolds [7]. Although polycaprolactone is widely used in medicine and has shown excellent potential in tissue engineering, as a hydrophobic material, its surface hydrophilicity is poor, which affects the adhesion and growth of cells on PCL scaffolds to some extent [8]. The elastic modulus of PCL may not match the elastic modulus of some soft tissues, which may result in poor mechanical matching between PCL scaffolds and surrounding tissues during soft tissue repair, thereby affecting the repair effect [9]. The application of single-polymer materials still has many limitations, and material properties can be optimized and adjusted through copolymerization or blending. Polyethylene glycol adipate (PEGA), as a derivative of polyethylene glycol (PEG), inherits the excellent biocompatibility of PEG [10]. PEG can quickly dissolve in tissue fluid in the body and be eliminated from the body without producing any side effects. Combining PEGA as a part of the material with PCL can endow biomaterials with new properties and functions, such as hydrophilicity and flexibility, which are of great significance for improving the performance of soft tissue biomaterials [11]. Polyurethane (PU), a polymer material with a shape memory effect, is formed by the condensation reaction of polyols and polyisocyanates. It has excellent biocompatibility, excellent physical and mechanical properties, and strong plasticity. Zhang et al. [12]. used PU combined with calcium phosphate to prepare scaffolds with enhanced mechanical properties and biocompatibility. There are various methods for preparing shape memory polyurethane scaffolds, including solvent casting/salting out, electrospinning, thermally induced phase separation, and gas foaming [13]. The gas foaming method prepares porous scaffolds with different pore structures and pore sizes by precisely controlling the rate and flow rate of gas escape. Research has shown that this method produces scaffolds with good connectivity, simple technology, and can effectively avoid pollution [14].

Using aliphatic hexamethylene diisocyanate (HDI) as the hard segment structure, polyethylene glycol adipate (PEGA)/polycaprolactone (PCL) porous tissue scaffold materials with five ratios of 7/3, 6/4, 5/5, 4/6, and 3/7 were prepared by the gas foaming method. A small amount of stannous octoate was used as a catalyst to promote the reaction and crosslink and polymerize the different properties of the soft and hard segments together to form polyurethane composite materials. We then tested the crystal structure, morphology, microstructure, thermal properties, functional groups, mechanical strength, and pore structure of the polyurethane composite materials to investigate the bone-repair ability of the PCL/PEGA polyurethane three-dimensional porous bone scaffolds.

### Preparation of PCL/PEGA Porous Scaffolds

**Figure 1:**
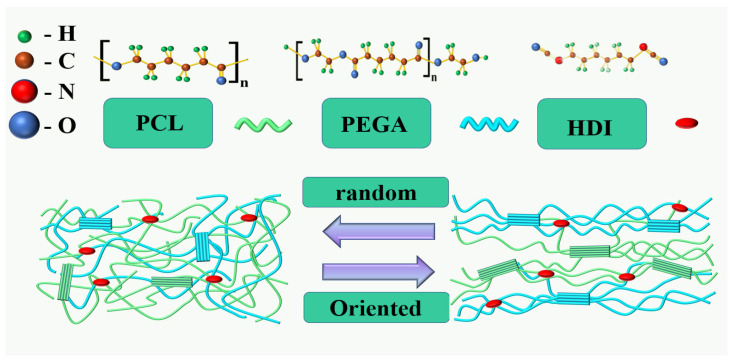
Raw materials and shape-changing mechanism diagram of 3D scaffold.

Firstly, weigh 20 g of polyethylene glycol adipate and 20 g of polycaprolactone corresponding to the composite ratio separately. Pour the weighed polycaprolactone into a beaker, seal it with cling film, and place it on a 90 °C stirrer. Stir at a speed of 200 r/min until completely dissolved, when the polycaprolactone solution will appear transparent. Next, pour the weighed polyethylene glycol adipate into the above-mentioned polycaprolactone solution according to the corresponding proportion. After the polycaprolactone solution is completely mixed with polyethylene glycol adipate, add 3.5 mL of hexamethylene diisocyanate (-OH:-NCO = 1:2~3) and five drops of tin octanoate, and continue the reaction for 2–5 h until the viscosity of the system increases. After the viscosity of the system has significantly increased, remove the cling film to completely evaporate the organic solvent, turning the system into a viscous melt. Add 2% deionized water by mass and mix evenly. Pour the mixed solution into a tin foil mold and place it in an oven at 120 °C to cure for 3 h. Finally, after the curing reaction is complete, cool it at room temperature to obtain a polyethylene glycol adipate/polycaprolactone porous tissue scaffold material (The chemical structures of raw materials and shape-changing mechanism diagram of 3D scaffold shown in Figure 1). According to different the copolymerization ratios of polycaprolactone and polyethylene adipate (PCL/PEGA 70/30, PCL/PEGA 60/40, PCL/PEGA 50/50, PCL/PEGA 30/70, PCL/PEGA 40/60), the bone scaffolds were named as PCL/PEGA 7/3, PCL/PEGA 6/4, PCL/PEGA 5/5, PCL/PEGA 4/6, and PCL/PEGA 3/7.

## 2. Materials and Methods

### 2.1. Materials

Poly(ε-caprolactone) ((PCL diol), M_n_ = 4000 g/mol) and Poly(ethylene glycol adipate) (PEGA, M_n_ = 2000 g/mol) were purchased from Jining Hua Kai Resin Co., Ltd., Jining, China. Hexamethylene diisocyanate (HDI, C_8_H_12_N_2_O_2_, >99.0%) and tin 2-ethylhexanoate (C_16_H_30_O_4_Sn, >95.0%) were obtained from Aladdin Chemical Reagent Factory, Shanghai, China.

### 2.2. XRD

The crystalline structure analysis was conducted using an X-ray diffractometer (XRD) (DX-2700, Fangyuan Instrument Co., Ltd., Dongguan city, China) operated at 40 kV and 25 mA [15]. The analysis was performed with Cu-Ka radiation in the 2θ range of 5–70° using a step size of 0.07° and a scanning speed of 0.3°/s.

### 2.3. SEM

The microstructure morphology of the composite materials was analyzed using a field-emission scanning electron microscope (FE-SEM, INSPECT F50, FEI, Eindhoven, Holland) at an accelerating voltage of 10 kV. Prior to analysis, the samples were coated with gold using a sputtering device [16].

### 2.4. FTIR

The functional groups of the PCL/PEGA were analyzed using a Fourier transform infrared spectrophotometer (FTIR, TENSOR-27, Bruker, Karlsruhe, Germany) in the wave number range of 400–4000 cm^−1^, with 64 scans per spectrum at a resolution of 4 cm^−1^.

### 2.5. Thermal Analysis Testing

Thermogravimetric testing was used to determine the thermal stability and decomposition process of the precursor, determine the heat treatment and carbonization temperature of the precursor, analyze the weight loss process, and discuss the mechanism of material formation. Under an air atmosphere, thermal stability testing was conducted using thermogravimetric analysis (TGA ST-A 409 PC, Shanghai Leiser Spectral Instrument Analysis Technology Co., Ltd., Shanghai, China) at a heating rate of 15 K/min from room temperature to 900 °C. By conducting thermal analysis tests on the prepared polycaprolactone/polyethylene adipate composite material, process changes such as loss on ignition, phase transition, and physical changes in the material were observed during the temperature increase. By conducting thermal analysis on composite materials with different proportions, it was possible to determine whether phase changes, thermal decomposition, thermal oxidation, and other chemical processes occurred in the composite materials during temperature changes.

### 2.6. Mechanical Performance Testing

The mechanical properties of the porous composite materials were determined using an electronic universal testing machine (MIT-30KN, Changzhou SFMIT Apparatus Co., Ltd., Guangzhou, China). This included assessing compression properties and Young’s moduli. Rectangular scaffold samples with dimensions of 10 mm × 10 mm × 10 mm were compressed until they reached approximately 60% of their original length.

### 2.7. Hydrophilicity and Gel Swelling Testing

Following the guidelines of ASTM standard D695–96, five scaffold samples with the same composition were tested at room temperature, 65% relative humidity (RH), and a crosshead speed of 1.0 mm·min^−1^ to obtain an average value. The density, porosity, and water absorption ratio of the porous materials were determined using a ceramic bulk density tester (DX-120C, Qunlong Instrument Co., Ltd., Xiamen, China) based on the Archimedes principle [17]. Three parallel samples were tested simultaneously to calculate average values. The results were calculated using the following formulas:

(1) $$\;\mathrm{Porosity}\;\left(\%\right)=\left(M_{3}-M_{1}\right)/\left(M_{3}-M_{2}\right)\times 100\%,$$

(2) $$\mathrm{Water}\;\mathrm{absorption}\;\mathrm{ratio}\;\left(\%\right)=\left[\left(M_{3}-M_{1}\right)/M_{1}\right]\times 100\%,\;$$

(3) $$\mathrm{Density}\;\left(g/{\mathrm{cm}}^{3}\right)=\left(M_{1}\times \mathrm{DL}\right)/(\left(M_{3}-M_{2}\right),\;$$

where $M_{1}$ is the dry weight of the porous composite materials determined in air; $M_{2}$ is the suspended weight of the anhydrous ethanol-impregnated porous composite materials; $M_{3}$ is the wet weight of the porous composite materials with saturated anhydrous ethanol states; and DL is the density of the saturated anhydrous ethanol.

The swelling behavior was evaluated by the change in volume. As-prepared cubic samples with diameters 10 mm × 10 mm × 10 mm (V_1_) were left to swell in deionized water and then cured in a refrigerator with at low temperature; subsequently, the volume (V_2_) was measured after the sample was vacuum-freeze-dried. The results were calculated by the following formula:

(4) $$\mathrm{Volume}\;\mathrm{change}\;\mathrm{ratio}\;\left(\%\right)=\left[\left(V_{2}-V_{1}\right)\;/{\;V}_{1}\right]\times 100\%,$$

In addition, PCL/PEGA porous scaffolds were cut into thin slices and used to test the wettability, and a minimum of 3 test pieces were used in each test. The water contact angle was measured by a contact angle meter (SDC 200, Yuding Precision Instrument Co., Ltd., Dongguan, China) [18].

### 2.8. Shape Memory Properties Testing

The molecular mechanism of shape memory in polymeric materials relies on the partial incompatibility among molecules within the polymer, enabling a reversible phase transition that allows the material to return to its original shape upon exposure to external stimuli while maintaining that shape otherwise. The fixed phase, characterized by a high thermal transition temperature in the PCL/PEGA polymer structure, plays a role in stabilizing the shape when deformation begins. When stimulated by external conditions, the molecular chains in the reversible phase rearrange, leading to a change in the shape of the PCL/PEGA. The melting temperature of polycaprolactone was used as the transition temperature for shape changing.

Initially, samples were prepared for the experiment, including cubic samples of 150 mm × 150 mm × 150 mm, rectangular samples of 150 mm × 10 mm, and star-shaped samples. Subsequently, the shapes of these samples were changed under external force, and then fixed at 0 °C. Photos were taken to record their fixed shapes, and the fixed angles of each sample were measured at 0 °C. The samples were then placed in an oven at 50 °C, the process of shape recovery was recorded with a camera, and we measured the bending angle of the sample at 90 s and 180 s. Finally, it is determined whether the composite material had shape memory performance and whether its shape memory performance was superior or inferior by comparing different sample angles at different times and calculating the shape recovery rate ($R_{r}$) was calculated using the following formula:

(5) $$R_{f}=\frac{\theta_{1}}{\theta_{0}}\times 100\%\;$$

where θ_0_ represents the initial angle (in degrees) and θ_1_ represents the recovered angle (in degrees) [19].

### 2.9. Statistical Analysis

For some data, multiple samples were selected for testing (n = 3). Data were expressed as the mean ± standard deviation (SD), and a *t*-test was performed to assess statistical significance. Statistical analysis was performed using GraphPad Prism 7.0 software. * represents the degree of significant difference.

## 3. Results

### 3.1. Fourier Transform Infrared Spectra (FTIR) Analysis of PCL/PEGA Porous Tissue Engineering Scaffolds

**Figure 2:**
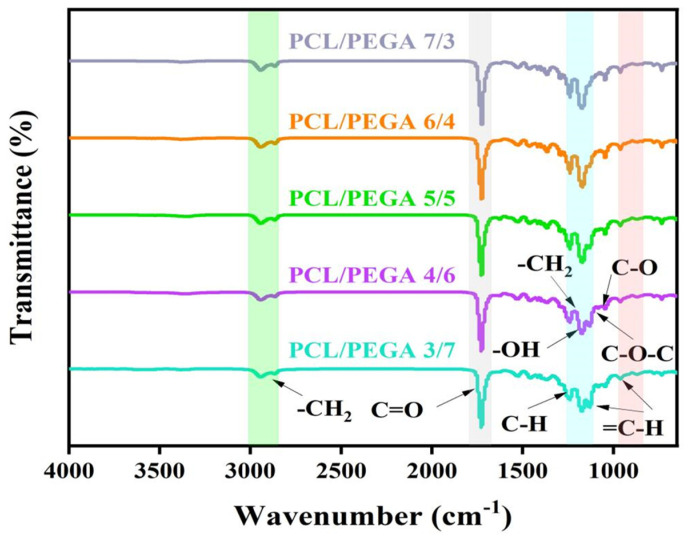
FTIR analysis of polycaprolactone/polyethylene adipate porous tissue scaffolds.

The FTIR of the PCL/PEGA porous tissue scaffolds with different proportions are shown in Figure 2. Distinct characteristic peaks were observed on the spectra, allowing for the determination of the characteristic functional groups of the PCL/PEGA composites and the intensity of each functional group. Both the polyethylene glycol adipate and polycaprolactone exhibit C-H vibration peaks at 2958 cm^−1^ and 2883 cm^−1^, which are caused by symmetric and asymmetric stretching vibrations of the methylene group. The characteristic peak at 1734 cm^−1^ is the absorption line of C=O stretching vibrations in the ester group. After the copolymerization of the two materials, the stretching vibration becomes more pronounced. The bending vibration spectrum of the methylene group is observed at 1333 cm^−1^, which is also evident after copolymerization. Subsequently, the C-O bond stretching vibration bands in the ester group are observed at 1258 cm^−1^ and 1167 cm^−1^, and the absorption peak of C-O-C appears at 1000–1055 cm^−1^. From the composite spectrum, it can be seen that the broad absorption band from 3319 cm^−1^ to 3530 cm^−1^ belongs to the N-H stretching absorption peak, and C-N stretching is observed at 1465 cm^−1^, indicating the formation of carbamate bonds during the reaction [20]. Hexamethylene diisocyanate can react with water to decompose. The absorption line of -NCO stretching vibrations at 2250 cm^−1^ does not appear in Figure 2, indicating that HDI has completely decomposed [21].

### 3.2. X-Ray Diffraction Analysis of PCL/PEGA Porous Tissue Engineering Scaffolds

**Figure 3:**
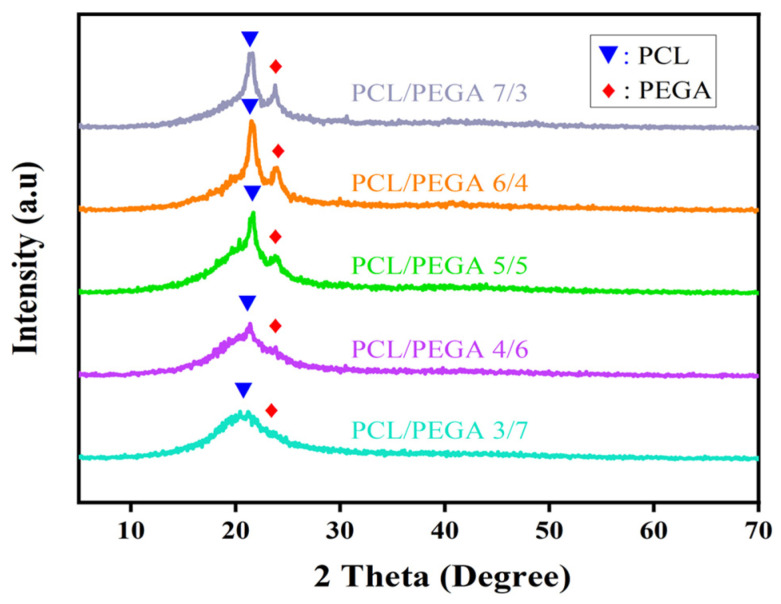
XRD of polycaprolactone/polyethylene adipate porous tissue scaffolds.

The X-ray diffraction spectra of the composites were compared with the PDF cards of polycaprolactone and poly(ethylene glycol adipate) to analyze whether there were changes in the phase composition of the composites. A horizontal comparison of the spectra of composites with different proportions was conducted to analyze whether there were differences in the material composition of the composites with varying proportions. Figure 3 shows the X-ray diffraction patterns of polycaprolactone/poly(ethylene glycol adipate) porous tissue scaffolds with different proportions. According to the literature [22], poly(ethylene glycol adipate) has three main diffraction peaks at 20.3°, 21.5°, and 24.2°, corresponding to the (111), (110), and (020) crystal planes, respectively. Pure polycaprolactone (PCL) exhibits diffraction peaks at 2θ values of 21.3°, 22.9°, and 23.6°, indicating that both pure polycaprolactone and poly(ethylene glycol adipate) have semi-crystalline structures [23].

### 3.3. Swelling Degree and Gel Content Analysis

PCL can be well dissolved in aromatic compounds, ketones, and polar solvents, and polyethylene glycol adipate is insoluble in water and easily soluble in acetone, toluene, ethyl acetate, and other organic solvents, so the caprolactone/polyethylene glycol adipate material was selected to be put into ethyl acetate to test its gel and swelling rate.

**Figure 4:**
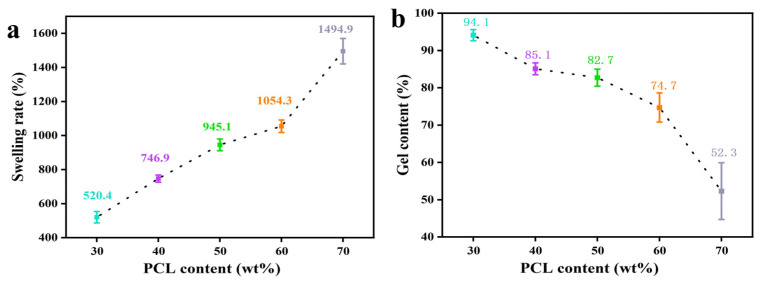
(**a**) Swelling degree of PCL/PEGA scaffolds; (**b**) gel content of PCL/PEGA scaffolds.

The swelling ratio and gel content of the polycaprolactone/polyethylene glycol adipate porous tissue scaffold are shown in Figure 4b.

As can be seen from the swelling ratio graph in Figure 4a, the volume change before and after freeze-drying after immersion in ethyl acetate ranges from 520.4% to 1494.5%. When the content of polycaprolactone is the lowest, the volume change is 520.4% [24]. As the content of polyethylene glycol adipate decreases and the content of polycaprolactone increases, the volume change rate increases significantly, indicating that the molecular chains in the scaffold stretch and expand in ethyl acetate, causing the original pore size to increase and the three-dimensional pore structure to become swollen. At the same time, after low-temperature treatment, it is fixed in an expanded state; after drying, the pore size contracts and becomes smaller, the ethyl acetate evaporates, the stretched and expanded molecular chains contract, and the sample becomes smaller and lighter.

From the change rule of the gel content of the scaffold materials in this figure, it can be concluded that with a decrease in polyethylene glycol adipate and an increase in polycaprolactone, the gel rate changes from 94.1% to 52.3%. After crosslinking, polycaprolactone and polyethylene glycol adipate not only maintain their superior properties, but also have good porosity, water absorption, flexibility, and certain mechanical properties, as well as shape memory properties. The gel ratio reflects the degree of crosslinking of polycaprolactone and polyethylene glycol adipate. The results show that the degree of crosslinking of the material is very good, and the degree of crosslinking decreases with the increase in polycaprolactone. It can be seen that polyethylene glycol adipate plays a major role in the crosslinking process. In addition to the effects of crosslinking time and temperature on the degree of crosslinking, the crystallinity, density and hydrogen bond of the crosslinked polymer have a great impact on the gel. It can be concluded that the higher the content of polyethylene glycol adipate, the better the degree of crystallinity and the better the degree of crosslinking.

### 3.4. Morphological Analysis of PCL/PEGA Scaffolds

**Figure 5:**
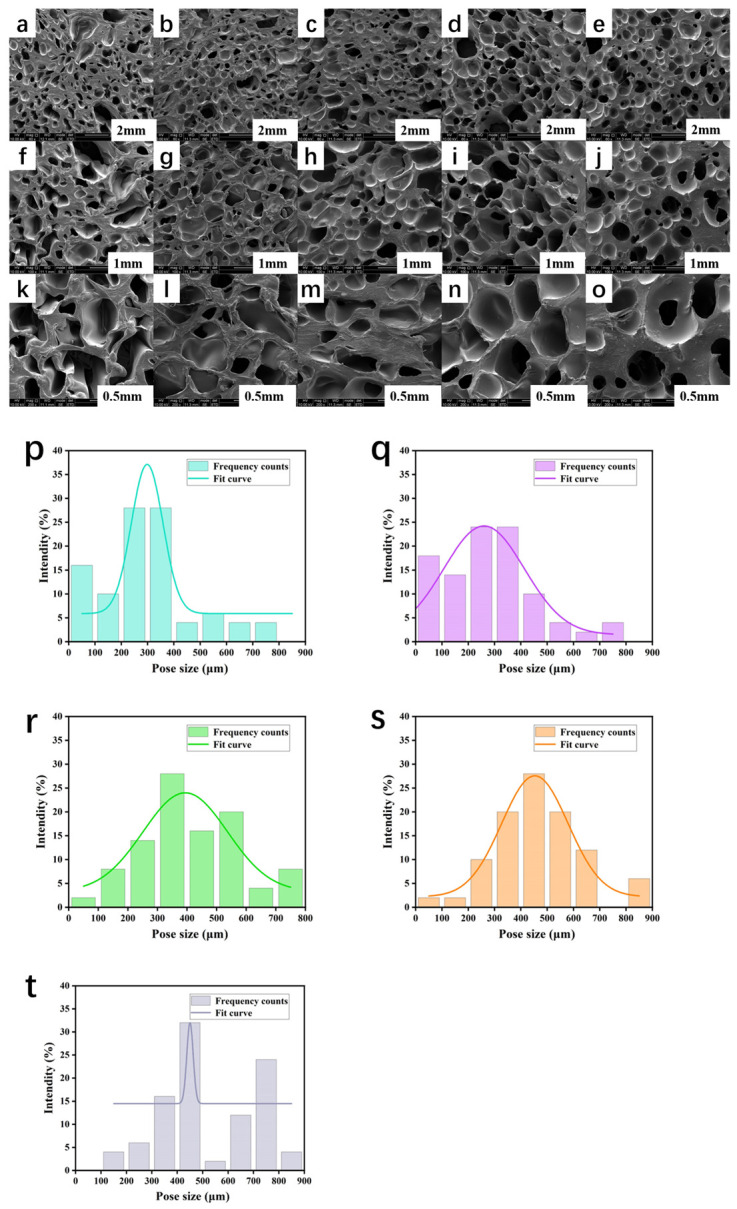
SEM images (**a**–**o**) and pore size distribution (**p**–**t**) of polycaprolactone/polyethylene adipate porous tissue scaffolds; PCL/PEGA-3/7 (**a**,**f**,**k**,**p**); PCL/PEGA-4/6 (**b**,**g**,**l**,**q**); PCL/PEGA-5/5 (**c**,**h**,**m**,**r**); PCL/PEGA-6/4 (**d**,**i**,**n**,**s**); PCL/PEGA-7/3 (**e**,**j**,**o**,**t**).

The scanning electron microscope (SEM) images of the morphology and pore size distribution of the PCL/PEGA porous scaffolds are shown in Figure 5. The images below are SEM images and pore size distribution diagrams at 60, 100, and 200 times magnification for different proportions. The figures show that all the scaffolds exhibit high porosity and relatively uniform pores. The pore size and porosity of the poly(ethylene glycol adipate)/polycaprolactone composite bone scaffolds increase with the increase in PCL content, and the pore size becomes more uniform. This indicates that the incorporation of polycaprolactone in the composite material provides excellent pore-forming properties. The pore size of the polycaprolactone/poly(ethylene glycol adipate) composite ranges from 100 to 200 μm for small pores, while the larger pores observed are distributed between 300 and 800 μm. The uniform pore size distribution indicates that the material has a continuous and excellent open pore structure. This suggests that the pore size distribution of the composite material is conducive to transporting nutrients and oxygen, enhancing cell viability, promoting metabolism and matrix vitality, and is suitable for promoting cell infiltration and adhesion, as well as providing a growth environment for new blood vessels [25].

### 3.5. Thermal Analysis of PCL/PEGA Scaffolds

Thermogravimetric analysis (TGA) was used to measure the thermal stability and decomposition process of the precursor, determine the heat treatment and carbonization temperatures of the precursor, analyze the weight loss process, and discuss the formation mechanism of the material [26]. By conducting thermal analysis tests on the prepared polycaprolactone/polyethylene glycol adipate composite materials, we can observe the materials’ burnout, phase transitions, physical changes, and other processes during temperature elevation. Through the thermal analysis of composite materials with different proportions, it can be determined whether there are phase changes in the composite material when the temperature changes, and whether chemical processes such as thermal decomposition and thermal oxidation occur, etc.

**Figure 6:**
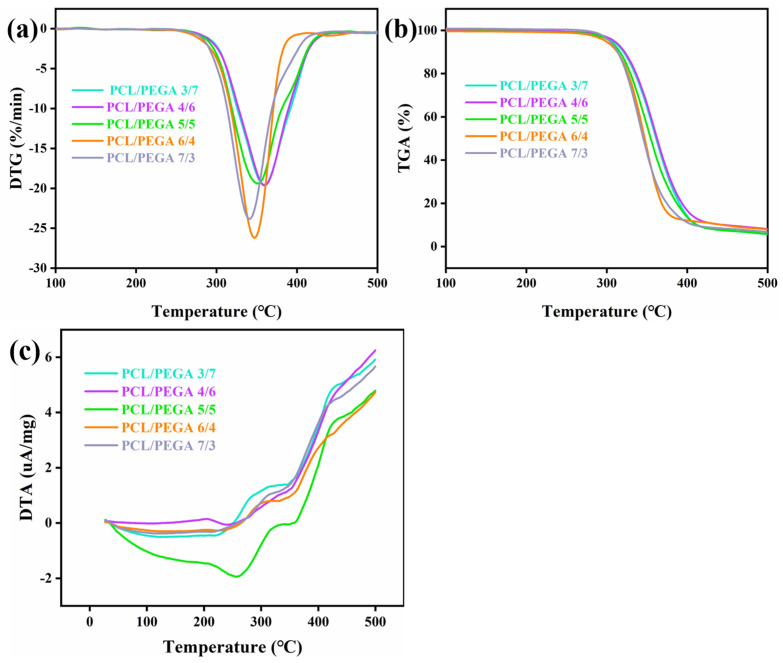
(**a**) DTG of polycaprolactone/polyethylene adipate porous tissue scaffolds; (**b**) TGA of polycaprolactone/polyethylene adipate porous tissue scaffolds; (**c**) DTA of polycaprolactone/polyethylene adipate porous tissue scaffolds.

The thermogravimetric analyses (TGA) of the polycaprolactone/polyethylene glycol adipate porous tissue scaffolds are shown in Figure 6a–c. The TGA curve includes three stages. In the first stage, when the temperature is around 100 °C to 280 °C, there is no change in weight, indicating no loss. The second stage is from 280 °C to 460 °C, during which a large amount of adsorbed water and decomposed bond groups in the porous cartilage tissue scaffold material of polycaprolactone/polyethylene glycol adipate are lost, accompanied by a significant amount of heat release, resulting in a weight loss rate of about 83%. The third stage is from 460 ° C to 500 ° C, with a residual mass of 17.6%. The previously decomposed bond groups and most of the adsorbed water have been lost, and the decrease in mass products here is due to the volatilization of the decomposition products of polycaprolactone and polyethylene glycol adipate.

The DTA curve can be divided into four parts, as per Figure 6c. First, there is an endothermic valley from about 37.5 °C to 280 °C, indicating a phase transition. During this process, the reaction tends to be stable without any phase changes, and the product starts to react endothermically. The second part is an exothermic valley between 280 °C and 340 °C, where a large amount of polycaprolactone/polyethylene glycol adipate decomposes and releases heat [27]. The next part is also a phase transition process from 340 °C to 460 °C. The last part is a small endothermic valley from 460 °C to 500 °C, where the decomposition products of polycaprolactone/polyethylene glycol adipate volatize.

For DTG, there is a significant peak at around 349 °C, indicating that the thermal decomposition rate of the polycaprolactone/polyethylene glycol adipate porous tissue scaffold material is the fastest at this temperature, and the material decomposes into carbon dioxide, acetaldehyde, etc. Analysis of the figure shows that as the content of polyethylene glycol adipate continuously increases, the initial endothermic temperature becomes higher, and the DTG peak value becomes lower. With the increase in polyethylene glycol adipate, the thermal decomposition rate decreases.

### 3.6. Porosity and Hydrophilicity of PCL/PEGA Scaffolds

**Figure 7:**
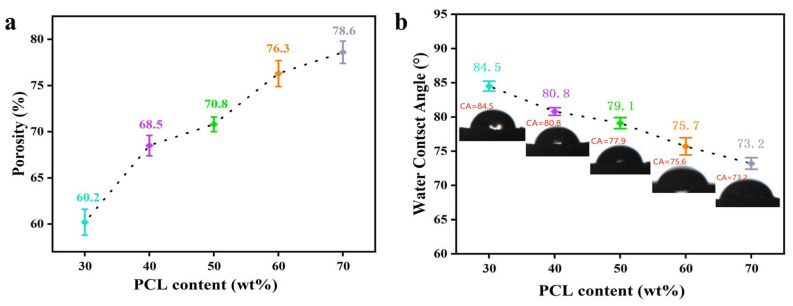
(**a**) Porosity of polycaprolactone/polyethylene adipate porous tissue scaffolds; (**b**) contact angles of polycaprolactone/polyethylene adipate porous tissue scaffolds.

The water absorption rate, porosity, and density of the polycaprolactone/polyethylene glycol adipate porous tissue scaffolds are shown in Table 1 and Figure 7a below. Table 1 presents the results of the data statistical analysis, expressed in the form of mean ± standard deviation.

According to Table 1, the water absorption rates of the PCL/PEGA scaffolds range from 176.5% to 234.6%, with porosity ranges from 60.2% to 78.6%. The porosity of the material is influenced by various factors such as the water (foaming agent) content, the proportion of isocyanate and PCL/PEGA, curing time, etc. From the changes in the different proportions of PCL/PEGA in Figure 7a, it can be concluded that polycaprolactone has better water absorbency compared to polyethylene glycol adipate [28]. Water can enter the copolymer and react with isocyanate, forming a three-dimensional porous tissue scaffold structure within the scaffold. In comparison, polyethylene glycol adipate has relatively poorer water absorbency. In summary, the polycaprolactone/polyethylene glycol adipate porous tissue scaffold has good porosity and water absorbency, which can promote cell migration, tissue ingrowth, and nutrient transport, while maintaining the basic mechanical properties required for life activities. Additionally, polycaprolactone content has a significant impact on water absorbency. The line graph of the average water contact angle of the polycaprolactone/polyethylene glycol adipate porous tissue scaffold and the photograph of the contact angle with deionized water are shown in Figure 7b. The data in the figure indicate that the average water contact angle of the polycaprolactone/polyethylene glycol adipate porous tissue scaffold decreases as the polycaprolactone content increases, indicating that an increased polycaprolactone content improves the wettability of the material.

### 3.7. Mechanical Properties of PCL/PEGA Scaffolds

**Figure 8:**
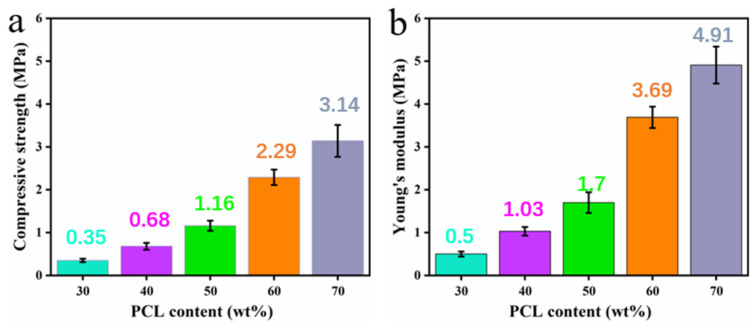
(**a**) Compressive strength of polycaprolactone/polyethylene adipate porous tissue scaffolds; (**b**) Young’s moduli of polycaprolactone/polyethylene adipate porous tissue scaffolds.

The mechanical properties of the polycaprolactone/polyethylene glycol adipate porous cartilage tissue scaffolds were tested using a hydraulic universal testing machine [29], as shown in Figure 8a,b.

There are many factors that affect the strength of polymeric materials. One category is the chemical and physical structure of the polymer, and the other category is external influencing factors such as temperature, deformation rate, pressure, etc. When external conditions are basically the same, the main factors affecting mechanical properties are the molecular weight and its distribution of the polymer, the conversion of OH groups, crystallization and orientation, substituents, copolymerization, etc. As illustrated in Figure 8b, as the content of polycaprolactone increases and the content of polyethylene glycol adipate decreases, the compressive strength gradually increases and the Young’s modulus also increases, showing a linear relationship between stress and strain. In summary, when the molecular content of polycaprolactone is higher than that of polyethylene glycol adipate, its mechanical properties increase. At the same time, as the amount of polycaprolactone increases and the conversion of OH groups of polyethylene glycol adipate improves, the strength increases. Moreover, this copolymer is successful and has good crystallization, which corresponds to the previous X-ray diffraction pattern. The mechanical properties of polyurethane are related to crystallinity, the ratio of hard and soft segments, and porosity. The porosity of the porous structure is inversely proportional to the mechanical properties.

### 3.8. Shape Memory Effect Analysis

**Figure 9:**
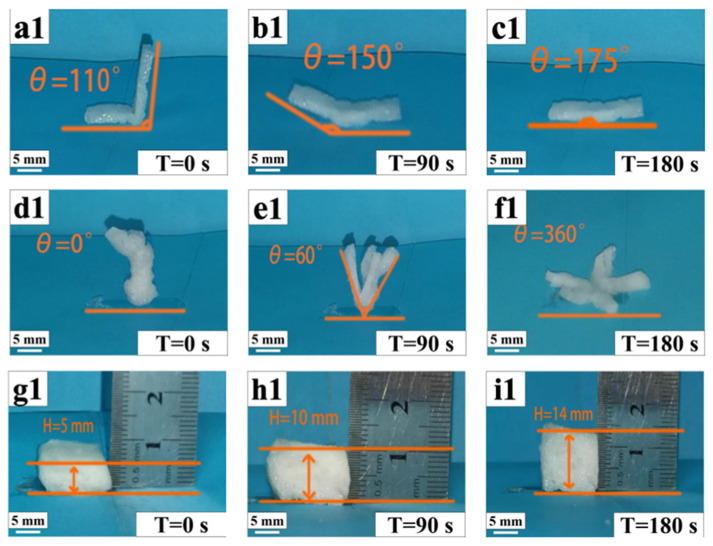
Square-shaped (**a1**–**c1**) and pentagonal star-shaped (**d1**–**f1**) scaffolds of polycaprolactone/polyethylene adipate porous tissue. Shape memory of cubic fast shapes (**g1**–**i1**).

From Figure 9, we can see that the strip-shaped, star-shaped, and cube-shaped structures all maintain a certain bending degree when cooled and fixed at 0 °C [30].

**Figure 10:**
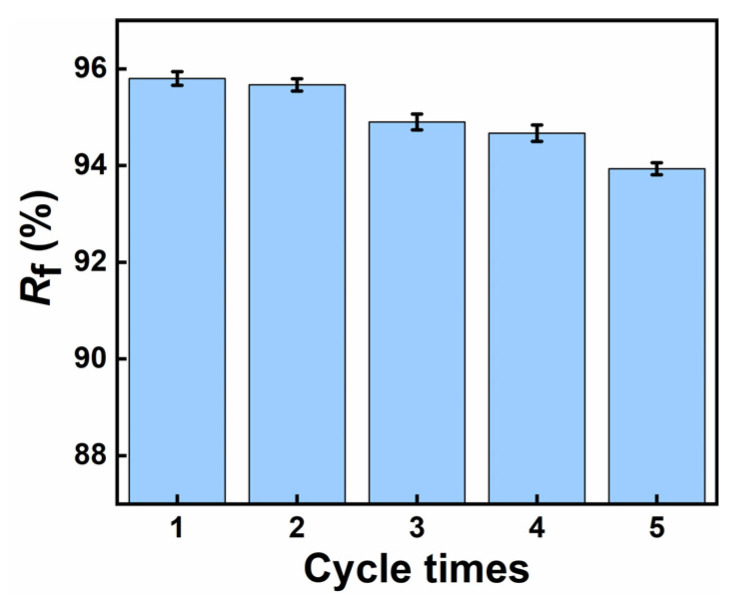
Deformation recovery rate Rf of polycaprolactone/polyethylene adipate porous tissue scaffold.

Then, at a melting temperature of around 50 °C, the shapes gradually recover. Ultimately, under continuous external temperature stimulation in the transition phase, the original shapes are restored. Figure 10 shows that the shape recovery rate of the five groups is approximately between 94 and 96%, demonstrating that the PCL/PEGA polymer has good shape memory properties.

## 4. Discussion

The in situ polymerization and simultaneous gas foaming process in this study successfully prepared a three-dimensional porous structure from PEGA and PCL with a porosity more than 60% and pore size ranging from 100 to 800 µm. According to the literature [31], within this range, cells have good growth space and can provide sufficient nutrients for themselves, facilitating the growth and adhesion of bone tissue cells. These morphological structures were confirmed by SEM results, showing that they could benefit the formation of bone-bonding interfaces and the growth of cells and tissues. All the IR and XRD analyses indicate that the PCL/PEGA composite is successfully prepared, in which the phase composition of both parts is stable, although the intensity of some peaks is influenced by the PCL content. Specifically, in the FTIR spectra, as the content of polyethylene glycol adipate increases, changes in peak intensity are observed from 1367 cm^−1^ to 1100 cm^−1^. The O-H vibration peak of polycaprolactone at 1367 cm^−1^ gradually disappears, while the bending vibration of the methylene group in polyethylene glycol adipate gradually intensifies. The diffraction patterns of XRD show that after the copolymerization of polycaprolactone and poly(ethylene glycol adipate), there is a broader diffraction band around 21°. The molecular weight of PCL is not high, and as the content of PCL increases, its crystallinity increases. This indicates that the polycondensation reaction between PCL and PEGA with diisocyanate in the soft and hard segments disrupts the original crystal structures of PCL and PEGA, reducing their mobility and crystallization ability [32].

From the changing gel content in the scaffold material in the figures, we can conclude that as the content of polyethylene glycol adipate decreases and the content of polycaprolactone increases, the gel content changes from 94.1% to 52.3%. The gel content reflects the degree of crosslinking between polycaprolactone and polyethylene glycol adipate. It can be seen from the figures that the material has a reasonable degree of crosslinking, but as the content of polycaprolactone increases, the degree of crosslinking decreases. Polyethylene glycol adipate plays a significant role in the crosslinking process. The crystallinity and density of the crosslinked polymer, as well as hydrogen bonding, significantly impact the gel (excluding the influence of polycondensation time and temperature on the degree of crosslink). It can be concluded that a higher content of polyethylene glycol adipate results in better crystallinity and a higher degree of crosslinking.

As is well known, the thermal degradation of PU mainly involves the decomposition reactions of different groups in its molecular chain. These groups include urethane, biuret, carbamate, and urea, which have different initial decomposition temperatures during thermal degradation. At high temperatures, these groups in the PU molecular chain undergo a series of complex chemical reactions, leading to the breakage of the molecular chain and the generation of degradation products. These degradation products may include isocyanates, alcohols, amines, carbon dioxide, etc. Comparing different proportions of polycaprolactone and polyethylene glycol adipate, a higher content of polycaprolactone results in a faster decomposition rate and higher burnout. For TGA curves corresponding to different proportions, a higher content of polycaprolactone results in greater curve fluctuations. Additionally, the final residual mass increases as the decomposition products decrease because the agglomeration of polycaprolactone prevents the volatilization of some of its decomposition products, thereby increasing the residual mass.

Compared to polyethylene glycol adipate, polycaprolactone has more hydrophilic groups and therefore exhibits better hydrophilicity. This scaffold material demonstrates that the crosslinking of polycaprolactone and polyethylene glycol adipate into a three-dimensional scaffold can better improve the hydrophilicity and water absorbency of the scaffold. The reason for this is that the hydrophilic groups of polycaprolactone are polar groups, which form hydrogen bonds in aqueous solutions or provide better wettability to hydrophobic groups in solute molecules. Therefore, as the polycaprolactone content increases, hydrophilicity increases.

Shape memory polymers (SMPs) are utilized in minimally invasive medical implant applications to reduce surgical trauma and avoid secondary damage, ultimately shortening the healing time [31]. In the initial heating phase, the reversible-phase (crystalline area) endothermic segment relaxes, and internal stress begins to release, leading to deformation. As the temperature reaches the melting temperature, the molecular segments become irregularly arranged, and the crystalline phase gradually transforms into a fully stress-relaxed state over time. The recovery speed of the stent increases with the PCL content in PCL/PEGA scaffolds, showing a clear difference. This is interpreted as PEGA hurting the regular arrangement of polyurethane molecule chains and reducing the crystallization performance of the scaffolds. As crystallization deteriorates, the melting temperature, which serves as the shape memory switch temperature, decreases. A lower enthalpy value for releasing internal stress results in faster shape memory recovery. The shape recovery rate slightly decreases with increasing cycle periods, interpreted as fatigue in the macroscopic polyurethane. When heated, the scaffold with a temporary shape restores to its initial state, releasing its porous structure and achieving a nearly 100% recovery rate. Correspondingly, the different shapes of the PCL/PEGA scaffold at 50 °C were recorded to clearly understand the morphological structure change during the shape recovery process (Figure 9). When the temperature reaches above Tm, the crystallization peak almost disappears, and the crystalline segments migrate into a disordered state. From the test results, it can be seen that the shape memory characteristics of the PCL/PEGA composite bone scaffold mean that it can reduce the trauma area when repairing bone defects and still be restored to its initial state after entering the body. PCL/PEGA composite bone scaffold, as a thermally responsive SMP material, can transform into any shape to repair bone defects when stimulated by human body temperature. At the same time, the material uses human body temperature as the transition temperature, which has a low impact on human tissue cells and tissue activity [33]. The transition temperature of the bracket is based on the melting temperature of the polymer, which has an endothermic valley between 37.5 °C and 280 °C. Shape recovery is a slow process involving the softening and migration of crystalline segments so that materials can recover below their peak values. So, the recovery shape is still uniformly heated in an environment of 37 °C. A few minutes of recovery time is acceptable, and a transitional period has been reserved for the surgery. At the same time, compared to other stimulus-responsive SMPS, the use and production of thermal-responsive SMPS are more convenient. The PCL/PEG composite bone scaffold prepared by Wang Li et al. showed excellent cell compatibility and osteoinductivity, with the authors detecting the cytotoxicity of the scaffold using CCK-8 and detecting the mineralization level of cells through qualitative and quantitative analyses of ALP expression levels [34]. Compared with scaffolds prepared from traditional materials (PCL or PEG), poly (ε-caprolactone) and polyethylene glycol composite scaffolds showed better biocompatibility [35]. PEGA has good biocompatibility as a derivative of PEG, so PEGA/PCL composite scaffolds also have good biocompatibility. The application of PCL/PEGA composite bone scaffolds has shown more feasibility for the medical application of SMPs, which can be used as minimally invasive bone tissue repair materials.

In addition, statistical analysis of test data during the experimental process, presented in the form of mean plus standard deviation (mean ± SD), can make the experimental test results clearer and more intuitive.

## 5. Conclusions

By employing the gas foaming method and utilizing different ratios of polyethylene glycol adipate (PEGA)/polycaprolactone (PCL) as porous tissue scaffold materials, we have successfully prepared bone scaffolds with good bone tissue properties. SEM imaging and pore size analysis results indicate that the PCL/PEGA materials prepared by this method have a high porosity with uniformly distributed pores, numerous interconnected pores, and pore diameters, mainly ranging from 100 to 600 μm. In the composite material, an increase in PCL content leads to an increased compressive strength and Young’s modulus, resulting in enhanced mechanical properties of the scaffold material, along with increased density and water absorption. XRD and thermal analysis results demonstrate that an increase in PEGA enhances the crystallinity of the polymer. In SEM scans and infrared spectroscopy, as the PEGA content decreases, porosity increases and flexibility improves. Under increased stress, the material does not break but only deforms, with pores in the porous material collapsing and the structure becoming denser. Additionally, the hydrophilicity of PEGA enhances the wettability of the polymer material. Finally, shape memory testing proves that the material exhibits an excellent shape memory effect, with a shape fixity rate above 93% and a shape recovery rate above 95%. In summary, the gas foaming method effectively prepares PCL/PEGA bone scaffolds, and this polymer possesses excellent bone tissue properties, holding great promise and potential research value for soft tissue applications.

## Figures and Tables

**Table 1:** Porosity, water absorption, and density of PCL/PEGA scaffolds.

Samples	Porosity (%)	Water Absorption (%)	Density (g/cm^3^)
PCL/PEGA 7/3	78.6 ± 4.2	234.6 ± 22.4	0.5 ± 0.01
PCL/PEGA 6/4	76.3 ± 4.9	214.8 ± 15.4	0.4 ± 0.03
PCL/PEGA 5/5	70.8 ± 3.2	199.6 ± 32.4	0.3 ± 0.01
PCL/PEGA 4/6	68.5 ± 3.5	180.3 ± 26.4	0.3 ± 0.02
PCL/PEGA 3/7	60.2 ± 1.8	176.5 ± 10.8	0.2 ± 0.03

## Data Availability

The original contributions presented in this study are included in the article. Further inquiries can be directed to the corresponding author.
